# Easy computation of the Bayes factor to fully quantify Occam’s razor in least-squares fitting and to guide actions

**DOI:** 10.1038/s41598-021-04694-7

**Published:** 2022-01-19

**Authors:** D. J. Dunstan, J. Crowne, A. J. Drew

**Affiliations:** School of Physics and Astronomy, Queen Mary University of London, London, E1 4NS UK

**Keywords:** Biological techniques, Biophysics, Computational biology and bioinformatics, Ecology, Medical research, Chemistry, Engineering, Materials science, Physics

## Abstract

The Bayes factor is the gold-standard figure of merit for comparing fits of models to data, for hypothesis selection and parameter estimation. However, it is little-used because it has been considered to be subjective, and to be computationally very intensive. A simple computational method has been known for at least 30 years, but has been dismissed as an approximation. We show here that all three criticisms are misplaced. The method should be used to complement and augment all least-squares fitting, because it can give very different, and better outcomes than classical methods. It can discriminate between models with equal numbers of parameters and equally good fits to data. It quantifies the Occam’s Razor injunction against over-fitting, and it demands that physically-meaningful parameters rejected by classical significance testing be included in the fitting, to avoid spurious precision and incorrect values for the other parameters. It strongly discourages the use of physically-meaningless parameters, thereby satisfying the Occam’s Razor injunction to use existing entities for explanation rather than multiplying new ones. More generally, as a relative probability, the Bayes factor combines naturally with other quantitative information to guide action in the absence of certain knowledge.

## Introduction

“If your experiment needs statistics, you ought to have done a better experiment.”—attributed to E. Rutherford. Nevertheless, almost every practising scientist, engineer, economist, etc., uses least-squares (LS) statistical methods to fit analytic expressions to data. This is done for parameter estimation (uncertainties as well as values) and for hypothesis or model selection^1^. However, LS fitting poses questions. How to know if the fit is as good as may be? How to choose between models which all fit well? How to detect over-fitting and under-fitting? These questions require quantitative tests based on statistical theory. There are well-known statistical tools—significance tests—such as the traditional *p* value or the 3 − σ test, and the more recent AIC and BIC (Akaike and Bayesian Information Criteria). Such tools are however inadequate, because they do not use the *prior* knowledge that we have^2^. The Bayes factor, derived from Bayes’ theorem, does do this, and so has been described as the gold-standard figure-of-merit for comparing models. However, it is rarely used, not least because it can be computationally very demanding. Here we present an easy way of calculating it so that it can be routinely used with all least-squares fitting to complement and augment other figures of merit. We demonstrate its use—and usefulness—on three datasets from the literature. Outcomes can be very different from those both of significance testing and of the BIC. Moreover, when considering not merely whether a theory—a model—is true or not, but, as a practical matter, deciding what action should be taken given the outcomes of the fitting, the Bayes factor can quantitatively support intuition.

Bayes’ Theorem explicitly includes prior knowledge in its calculation of the probability of a hypothesis given data. It was an unexceptionable part of probability theory in the nineteenth century. However, the increasing formalisation of probability theory and statistics in the twentieth century led to its sidelining, on the grounds that it introduces a subjective element, our state of knowledge, or grounds for belief, about future events. It was considered that probabilities should be purely objective. Jeffreys’ seminal book in 1939 began the rehabilitation of Bayesian statistics^3^. This has been slow and controversial. For an entertaining historical survey, see the article by Leonard^4^, and for a non-technical discussion see Jaynes^5^. For an early technical account, see Kass and Raftery^6^.

Occam’s Razor (“Entities should not be postulated without necessity”), in the context of least-squares fitting, demands that we should not use more fitting parameters than are necessary. That is, we should not overfit data. Classical—twentieth century—statistics scarcely quantifies this. In 1974, Akaike introduced the AIC to quantify this issue by preferring the model with the highest log-likelihood [see Eq. (1) in "Methods" section] less a penalty of *n*, the number of parameters^7^. The AIC can be applied directly to least-squares fitting, where the preferred model is the one with the lowest sum of the squares of the residuals (the SSR) after applying the AIC penalty. The AIC has now been largely supplanted by the BIC or Schwartz criterion^8^ (SBIC), like the AIC except that the penalty for *n* parameters is ½*n*ln*m* where *m* is the number of data points. (Both the AIC and the BIC are usually presented after multiplication of these definitions by − 2; we do not do that here, to make comparison of the figures of merit easier. We indicate this by referring to the SCBIC.) The BIC is now widely used^9–11^. Thousands of papers per year now cite BIC values to justify the choice of one model rather than another, e.g. in ecology^12^. However, the AIC and the BIC and many related criteria (DIC, FIC ... WAIC) are gross approximations to the Bayes factor. Indeed, despite its name, the BIC is not Bayesian, and nor are the various related criteria. This is because they do not take into account the *prior* probabilities of the models. The Bayes factor does. In so doing, it quantifies two further intuitions, or corollaries, of Occam’s razor. The first is that fits to data that use physically-meaningful parameters are preferable, if they fit, to fits that use physically-meaningless parameters such as coefficients in a polynomial or Fourier series. The latter introduce new entities while the former use entities that already exist. The second, closely related intuition, is that a model that is not capable of fitting all possible datasets (that does not span the data space) yet does fit the actual dataset is preferable to a model that could fit any data presented (that does span the data space).

Despite being the gold standard, the Bayes factor is little known and less used. It has been considered to be computationally massively intensive^6,8–11^. Except in simple problems of models with one fitting parameter, evaluating the Bayes factors of the models has required multi-dimensional integrals over parameter space. Fitting, for example, a multi-peak spectrum with tens of parameters, this requires computationally-heavy techniques such as Markov-chain Monte-Carlo integration or related sampling methods (see, e.g. Gronau et al.^13^). Because of the taint of subjectivism, in its use of what we know, many Bayesians have preferred to avoid prior knowledge and use in its place information obtained from the data, such as unit information priors^6,14,15^. Yet this concern is misplaced. What we know before analysing data is as objective, in the usual scientific sense, as the data themselves.

Here we present a formula for easy calculation of the Bayes factor after every LS fit with much less computational effort than the fit itself. This formula has been known since at least 1992^15^, and perhaps earlier. Its use in routine LS fitting has not been widely advocated. This is perhaps because of the subjectivity issue, or perhaps because it bypasses the computational difficulties of the Bayes factor by the Laplace approximation^16^. However, McKay already in 1992 recognised this as exact in most situations^15^. Perhaps also there has not been sufficient appreciation of the value of the Bayes factor in quantifying the two further intuitions of Occam’s razor mentioned above, and its value as a guide to action. We present the method in "Methods" section. In "Theory" section we briefly discuss the underlying theory, and in the Supplementary Information (SI §5) we give a derivation of the formula which we hope makes the underlying ideas clearer than they were in the older literature. In "Examples of fitting data" section we apply it to three examples of data-fitting in which the use of the Bayes factor leads to very different—and better—outcomes than traditional methods. Finally, in "Discussion and conclusions" section, we discuss the main outcomes, and consider the relevance of the Bayes factor to two live controversies. On significance (*p* values etc.) in fitting, we find that reliance on significance and the rejection of physically-meaningful parameters that do not pass significance tests will normally give incorrect results. On the controversial question of vitamin D and Covid-19, there is evidence that does not pass significance tests. We see how the Bayes factor can combine with this evidence to provide quantitative support for actions that otherwise are considered unjustified.

## Methods

A least-squares fitting routine normally returns the parameter fitted values and their uncertainties, the fit residuals *r*_*i*_ and their standard deviation σ_*r*_, and perhaps the parameter covariance matrix **Cov**_**p**_, the BIC, etc. The formula we apply uses the marginal likelihood integral (MLI) calculated for each LS fit. See "Theory" section. Calculating the MLI is done by,

1 $${\text{MLI}} = \left( {2\pi } \right)^{n/2} L_{max} \frac{{\sqrt {{\text{det }}{\mathbf{Cov}}_{{\varvec{p}}} } }}{{\mathop \prod \nolimits_{i = 1}^{n} {\Delta }p_{i} }}$$

where *n* is the number of parameters, the ${{\varvec{\Delta}}}{\varvec{p}}_{{\varvec{i}}}$ are their ranges, and *L*_*max*_ is the maximum likelihood^15^. Then the Bayes factor between two models is the ratio of their MLI values. The first step in applying it is to calculate *L*_*max*_, which is the value of the likelihood *L* at the fitted parameter values whether LS or ML fitting is used. *L* is the product of the probability densities of all the *m* datapoints given the fit. If it is not returned by an LS routine, it is readily calculated (see SI §S2). With perhaps hundreds of datapoints, *L* can be a very large or a very small number, depending on the value of the standard deviation of the residuals, σ_*r*_, so it is more convenient to work with the log-likelihood, ln*L*. Equation (S1) in the SI shows that for a Gaussian distribution of residuals, maximising ln*L* is equivalent to minimising the SSR. If the LS routine returns the SSR, then it is particularly easy to calculate ln*L*.

Next, we need **Cov**_**p**_. With software such as Mathematica, Matlab, or Origin, this is returned by the LS routine. If it has to be calculated, we show how in SI §S3.

The remaining term in Eq. (1) is the product of the *n* parameter ranges, ${\Delta }p_{i}$, which gives the prior probability of the model. The ranges have to be decided upon and input by the user. There is nothing subjective about this, determined as they should be objectively (or evidentially) by our *prior* scientific knowledge. They are open to reasoned debate and justification like any scientific knowledge or data. See SI §S4, and the examples in "Examples of fitting data" section.

When we have the MLI values for two or more fits, their ratios give the relative probabilities for the models given the data—the Bayes factors (BF) between the models. It is more convenient to work with the logarithms, and then it is the difference of the lnMLI values, lnBF, which matters. Jeffreys^3^ and many subsequent authors have given verbal descriptions of the meaning of values of lnBF, in terms of the strength of the evidence in favour of the model with the higher lnMLI. These range from lnBF < 1—barely worth considering, 1–2—substantial, 2–5—strong evidence, > 5—decisive^3,6^. More important than the verbal descriptions is that the Bayes factor simply expresses the relative probabilities of the models. The lnBF values correspond to odds of *e*^lnBF^ to 1 on the preferred model, or against the other model. The descriptions and the odds also apply to comparing models by differences in ln*L*_*max*_ between models with the same of parameters, and by the Schwartz BIC (SBIC = − ½BIC, which we use here for easy comparison with ln*L*, lnMLI and lnBF). It is important to note that debate or dispute over the exact values of the parameter ranges is rarely germane, since it is their logarithms that enter into the lnBF. So a factor of two on a range shifts the lnBF by only ln2, or 0.7.

## Theory

Equation (2) for the marginal likelihood integral has been given by many authors. Following Gull^17^ we consider it first for a problem involving just one parameter λ distinguishing two versions of a theory (*The Story of Mr A and Mr B*, proposed originally by Jeffreys^3^ and discussed by many authors). Mr A advocates the null hypothesis, **A,** in which this parameter does not appear. Mr B advocates the hypothesis, **B**, in which λ appears; least-squares fitting to the data **D** yields the fitted value λ_0_ ± δλ. Occam’s razor tells us that the extra parameter λ should only be included if it is necessary. Then Bayes’ theorem gives for the value of the Bayes factor, BF, for B against A,

3 $${\text{BF}} = \frac{{Pr\left( {\text{B|D}} \right)}}{{Pr\left( {\text{A|D}} \right)}} = \frac{{Pr\left( {\text{B}} \right)}}{{Pr\left( {\text{A}} \right)}} \times \frac{{Pr\left( {{\text{D|B}},\uplambda _{0} } \right)}}{{Pr\left( {\text{D|A}} \right)}} \times \frac{{\sqrt {2\pi } \updelta \uplambda }}{{\uplambda _{\max } -\uplambda _{\min } }}$$

where Gull explains the first term in the RHS, *Pr*(**B**)/*Pr*(**A**), as having nothing to do with the theories or the data; it will normally be unity. Perhaps slightly tongue-in-cheek, Gull proposed that it could be adjusted to reflect the past performances of Mr A and Mr B. We take this term as unity here but we return to it in "Discussion and conclusions" section. The second term in the RHS is the ratio of the maximum likelihoods (or of the SSRs from LS fitting), which will normally favour **B** because adding fitting parameters will normally improve the fit to data. For **B**, it is the likelihood evaluated at the fitted value, λ_0_. The third term in the RHS is the Occam factor, which will provide the penalty for the extra parameter in **B**. As Gull explains it, Mr B had to spread his probability *Pr*(**B**) over the *prior* range that he will have specified of possible values of λ from λ_min_ to λ_max_, with some pdf, that is usually assumed to be flat from λ_min_ to λ_max_ and zero elsewhere^6,15,17^. When the data are given, the probability of the model becomes the integral (the MLI) of the product of this pdf and the function *L*(λ). Most of these possible parameter values perish and only a range around the fitted value λ_0_ survive. The shape of *L*(λ) around is approximated by a Gaussian. The width of this Gaussian, σ_λ_, is the uncertainty or error δλ returned by the LS routine for λ^2,17^. This is the Laplace approximation^6,14,15^. Evaluation of the integral thus requires no more than taking the area of the Gaussian times the flat value of Mr B’s prior pdf, 1/(λ_max _− λ_min_).

For models differing from the null hypothesis in more than one extra parameter, one might think that Eq. (3) could be generalised by multiplying the Occam’s factors (the third term) for all the extra parameters together. That, however, normally grossly overestimates the MLIs, because of correlation or covariance between the parameters in the fits. The remedy is to use the square-root of the determinant of the parameter covariance matrix in place of the product of the uncertainties of the fitted parameter values, as in Eq. (1). This is again the Laplace approximation; see SI §5 for an explanation.

The ranges define a volume in the *n*-dimensional parameter space, known as the prior parameter volume. Similarly, the square-root of the determinant of the covariance matrix defines another, smaller volume in the same space, the posterior parameter volume. The ratio of these two volumes is termed the Occam Factor^17–19^.

Our Eq. (1) is well-known in the literature, for example, it is Eq. (6) of MacKay’s 1992 paper^15^, Eq. (10.123) of Gregory’s 2005 book^20^. And Eq. (4.137) of Bishop’s 2006 book^21^. However, in the rest of McKay’s paper and in most of the subsequent literature, the prior parameter volume in the denominator is not determined from our knowledge of the parameters and what values are physically realistic. Instead, it is determined from the data and the outcome of the fit, the posterior parameter distributions (e.g. unit information priors). Indeed, that is the key step in using Eq. (1) to derive the BIC^15,20^, and is the reason the BIC treats all parameters alike. Gull^17^ discusses the selection of the volume in the special case of one fitting parameter only, where the covariance matrix is not needed. Sivia and Skilling^2^ also consider it but in the context of maximum likelihood fitting and apparently much more complicated calculations, in which our Eq. (1) is their Eq. (4.20). Much of the discussion of choice of priors is on mathematical, not physical grounds^13,20–22^. For a very recent survey, see Rougier and Priebe^19^. The SI §5concludes with a comparison of what we do here and what is standard in the Bayesian literature.

It is worth noting that Eq. (1) is never analytically exact, because of the truncation of the integrals of the Gaussian functions *L*(*p*_*i*_) at the edges of the parameter prior volume, and eventually if *L*(*p*_*i*_) are not Gaussians. It is not difficult to check whether these issues are significant, nor to make reasonable corrections to the MLI when they are. See Example 3 in "Background in fitting a carbon nanotube Raman spectrum" section, and SI §8Fig. S3.

These methods are applicable to Maximum Likelihood (ML) fitting. In contrast to LS fitting, ML fitting can easily handle the simultaneous fitting of multiple data sets, and datasets with different uncertainties σ_*i*_ on different residuals *r*_*i*_, and it can handle outliers in a rigorous and respectable way^23–25^. See Example 2 ("Discriminating between models for the pressure dependence of the GaAs bandgap" section and SI §7) for both these issues.

## Examples of fitting data

### How many parameters best describe data in muon spectroscopy?

**Figure 1:**
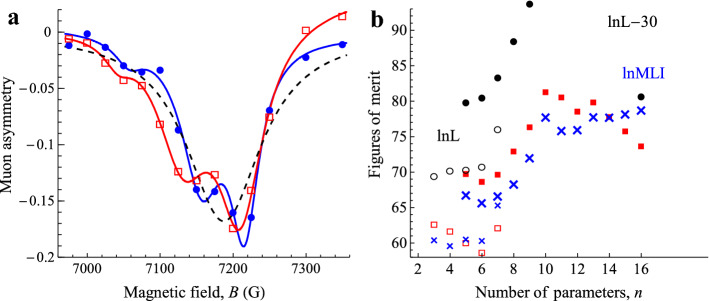
Muon-spin spectroscopy. Data from an experiment, muon polarisation as a function of magnetic field^27^, is shown in (**a**). Error bars on the data are estimated at ± 0.015. Linear background functions due to positrons have already been subtracted from the data. The blue solid-circle datapoints () were recorded in the dark, while the red open-square datapoints () were photo-excited. The blue and red solid lines show 19-parameter fits of three Lorentzian peaks and two linear backgrounds, separately for the data in the dark (bluesolid line) and photo-excited (red chain-dotted line). In (**b**), the evolution of the figures of merit of the fit with the number *n* of fitting parameters is shown (  SBIC, lnMLI,  ln*L* with the last four points shifted down by 30). The open or small data points from three to seven parameters are for a single peak. The solid or large datapoints from five to 16 parameters are for two peaks, and from 17 to 20 parameters for three peaks. (Figure prepared using Mathematica 12.0, www.wolfram.com/mathematica/).

Here we find that the Bayes factor demands the inclusion of more physically-meaningful parameters than the BIC or significance tests. Figure 1a presents some data that might reasonably be fitted with as few as three or as many as 22 physically-meaningful parameters. We find that the Bayes factor encourages the inclusion of all these parameters until the onset of over-fitting. Even though many of them have fitted values that fail significance tests (i.e. are consistent with zero), their omission distorts the fitting results severely.

Figure 1a shows an anti-level-crossing spectrum observed in photo-excited muon-spin spectroscopy^26^ from an organic molecule^27^. The data are presented in Fig. 2a of Ref.^27^ and are given in the SI. These spectra are expected to be Lorentzian peaks. Theory permits optical excitation to affect the peak position, the width and the strength (photosensitivity). In the field region over which the measurements are carried out, there is a background from detection of positrons, which has been subtracted from the data presented^27^. Wang et al.^27^ did not attempt to fit the data rigorously; they did report a model-independent integration of the data, which demonstrated a change in area and position.

The model that we fit hypothesises one or more Lorentzian peaks, with optional photosensitivity on each fitting parameter and with optional linear backgrounds *y* = *a* + *bx* underlying the peaks, described by the full equation given in the SI, equation (S3). To do a single LS fit to all the data, we extend the data to three dimensions, (*x* gauss, *y* asymmetry, *z*) where *z* = 0 for data in the dark and *z* = 1 for photoexcited data. Including all the data in a single LS fit in this way, rather than fitting the dark and photoexcited data separately, simplifies both setting up the fit and doing the subsequent analysis.

Figure 1b shows the evolution of the SBIC and the lnBF as the number of fitting parameters in the model is increased. Starting with a single Lorentzian peak, three parameters are required, peak position *P*, width *W* and intensity *A.* Three photosensitivity parameters Δ_L_*P*, Δ_L_*W* and Δ_L_*A* are then introduced successively to the fit, (open and small data points for *n* = 3–6). The SBIC decreases and the lnMLI scarcely increases. It is only with the inclusion of one background term (*n* = 7) that any figure of merit shows any substantial increase. There is no evidence here for photosensitivity. The weak peak around 7050 G does not seem worth including in a fit, as it is evidenced by only two or three data points and is scarcely outside the error bars. However, a good fit with two peaks (*P*_1_ ~ 7210 G, *P*_2_ ~ 7150 G, the subscripts 1 and 2 in accordance with the site labelling of Fig. 2a of Ref.^27^) can be obtained with just five parameters (*P*_1_, *P*_2_, *A*_1_*, A*_2_, *W*). This gives substantial increases in the SBIC and lnMLI, further increased when *W*_1_ and *W*_2_ are distinguished and then when the single background term and the three photosensitivity parameters Δ_L_*P*_*2*_, Δ_L_*W*_2_ and Δ_L_*A*_*2*_ are successively included (solid or large data points for *n* = 5–10 in Fig. 1b). The SBIC reaches its maximum here, at *n* = 10, and then decreases substantially when the other three photosensitivity parameters and the other three background terms are included. These additional parameters fail significance tests as well as decreasing the SBIC (Fig. 1b). Conventionally, the *n* = 10 fit would be accepted as best. The outcome would be reported as two peaks, with significant photo-sensitivities Δ_L_*P*_2_, Δ_L_*W*_2_ and Δ_L_*A*_2_ for all three of the 7150 G peak parameters, but no photosensitivity for the 7210 G peak (Table 1).

**Table 1:** Photosensitivity results of fitting the data of Fig. 1a with 10, 16 and 19 parameters. Parameter units as implied by Fig. 1a.

	Δ_L_*P*_1_	Δ_L_*W*_1_	Δ_L_*A*_1_	Δ_L_*P*_2_	Δ_L_*W*_2_	Δ_L_*A*_2_
10-parameter fit	–	–	–	− 14 ± 4 *p* = 0.002	21 ± 6 *p* = 0.003	− 9 ± 2 *p* = 0.0002
16-parameter fit	− 5 ± 3.5 *p* = 0.18	12 ± 8 *p* = 0.16	− 7 ± 5 *p* = 0.20	− 24 ± 8 *p* = 0.008	16 ± 13 *p* = 0.24	− 5 ± 6 *p* = 0.45
19-parameter fit	− 6 ± 2.8 *p* = 0.07	14 ± 6.2 *p* = 0.05	− 9 ± 3.8 *p* = 0.05	− 25 ± 4.8 *p* = 0.0006	10 ± 9 *p* = 0.3	− 2.3 ± 4 *p* = 0.6

The Bayes factor gives a very different outcome. From 10 to 16 parameters, the Bayes factor between any two of these seven models is close to unity (Fig. 1b). That is, they have approximately equal probability. The Bayes factor shows that what the conventional *n* = 10 analysis would report is false. Specifically, it is not the case that Δ_L_*P*_2_, reported as − 14 ± 4 G, has a roughly $${\raise0.5ex\hbox{$\scriptstyle 2$} \kern-0.1em/\kern-0.15em \lower0.25ex\hbox{$\scriptstyle 3$}}$$ probability of lying between − 10 and − 18 G. That is not consistent with the roughly equal probability that it lies in the *n* = 16 range (− 24 ± 8 G). Table 1 shows that at *n* = 16, Δ_L_*P*_2_ is the only photosensitivity parameter to pass significance tests. Δ_L_*A*_2_, which had the highest significance level at *n* = 10, is now the parameter most consistent with zero. The other four are suggestively (about 1$${\raise0.5ex\hbox{$\scriptstyle 1$} \kern-0.1em/\kern-0.15em \lower0.25ex\hbox{$\scriptstyle 2$}}$$σ) different from zero.

Since the Bayes factor has already radically changed the outcome by encouraging more physically-meaningful parameters, it is appropriate to try the 7050 G peak parameters in the fit. With only 28 data-points, we should be alert to over-fitting. We can include *P*_3_ and *A*_3_ (*n* = 18), and Δ_L_*P*_3_ (*n* = 19), but *W*_3_ and Δ_L_*A*_3_ do cause overfitting. Figure 1b shows substantial increases of both the SBIC and the lnMLI for *n* = 18 to *n* = 20, where the twentieth parameter is in fact Δ_L_*A*_3_. The symptom of over-fitting that we observe here is an increase in the logarithm of the Occam Factor (lnMLI − ln*L*), the values of which decrease, − 26.9, − 33.5, − 34.8, and then increase, − 33.4, for *n* = 16, 18, 19 and 20 respectively. Just as ln*L* must increase with every additional parameter, so should the Occam factor decrease, as the prior parameter volume should increase more with a new parameter than the posterior parameter volume. So we stop at *n* = 19. The outcome, Table 1, is that the uncertainties on the *n* = 16 parameters have decreased markedly. This is due to the better fit, with a substantial increase in ln*L* corresponding to reduced residuals on all the data. The 7210 G peak 2 now has photosensitivities on all its parameters, significant to at least the 2σ or *p *value ~ 0.05 level. And the photosensitivities Δ_L_*W*_2_ and Δ_L_*A*_2_, both so significant at *n* = 10, and already dwindling in significance at *n* = 16, are both now taking values quite consistent with zero. In the light of Table 1, we see that stopping the fit at *n* = 10 results in completely incorrect results—misleading fitted values, with certainly false uncertainties.

### Discriminating between models for the pressure dependence of the GaAs bandgap

The main purpose of this example is to show how the Bayes factor can be used to decide between two models which have equal goodness of fit to the data (equal values of ln*L* and BIC, as well as *p* values, etc.). This illustrates the distinction it makes between physically-meaningful and physically meaningless parameters. This example also shows how ML fitting can be used together with the Bayes factor to obtain better results. For details, see SI §7.

**Figure 2:**
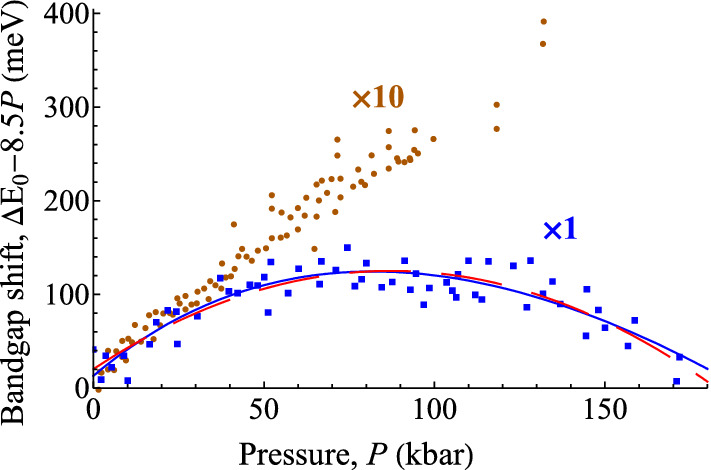
GaAs band-gap. Data for *E*_*g*_(*P*) in GaAs from Goñi et al*.*^28^ ( ) and from Perlin et al*.*^29^ ( ) are shown after subtraction of the straight line *E*_0_ + 8.5*P* to make the curvature more visible. The Perlin data is expanded × 10 on both axes for clarity. Two least-squares fits to the Goñi data are shown, polynomial (dashed red line) and Murnaghan (solid blue line). (Figure prepared using Mathematica 12.0, www.wolfram.com/mathematica/).

Figure 2 shows two datasets for the pressure dependence of the bandgap of GaAs (data given in the SI). The original authors published quadratic fits, ${E}_{g}(P)={E}_{0}+bP+c{P}^{2}$, with *b* = 10.8 ± 0.3 meV kbar^−1^ (Goñi et al*.*^28^) and 11.6 ± 0.2 meV kbar^−1^ (Perlin et al.^29^). Other reported experimental and calculated values for *b* ranged from 10.02 to 12.3 meV kbar^−1^^30^. These discrepancies of about ± 10% were attributed to experimental errors in high-pressure experimentation. However, from a comparison of six such datasets, Frogley et al.^30^ were able to show that the discrepancies arose from fitting the data with the quadratic formula. The different datasets were reconciled by using the Murnaghan equation of state and supposing the band-gap to vary linearly with the density (see SI, §7, equations (S4) and (S5)^30^. The curvature *c* of the quadratic is constant, while the curvature of the density, due to the pressure dependence *B*ʹ of the bulk modulus *B*_0_, decreases with pressure—and the six datasets were recorded over very different pressure ranges, as in Fig. 2. So the fitted values of *c*, *c*_0_, were very different, and the correlation between *b* and *c* resulted in the variations in *b*_0_.

Here, using the Bayes factor, we obtain the same result from a single dataset, that of Goñi et al.^28^ The two fits are shown in Fig. 2. They are equally good, with values of ln*L* and SBIC the same to 0.01. The key curvature parameters, *c* and ${\text{B}}^{\prime }$, are both returned as non-zero by 13.5σ (SI, §7, Table S1), consequently both with *p*-values less than 10^−18^. However, *c* is a physically-meaningless parameter. The tightest constraint we have for setting its range is the values previously reported, ranging from 0 to 60 μeV kbar^−2^, so we use Δ*c* = 100 μeV kbar^−2^. In contrast, ${\text{B}}^{\prime }$ is known for GaAs to be 4.49^31^. For many other materials and from theory the range 4–5 is expected, so we use $\Delta {\text{B}}^{\prime } = 1$. The other ranges are same for both models (see SI §7). This difference gives a lnBF of 3.8 in favour of the Murnaghan model against the quadratic, which is strong evidence for it. Moreover, the value of ${\text{B}}^{\prime }$ returned is 4.47 ± 0.33, in excellent agreement with the literature value. Had it been far out of range, the model would have to be rejected. The quadratic model is under no such constraint; indeed, a poor fit might be handled by adding cubic and higher terms *ad lib*. This justifies adding about 5 to lnBF (see "Background in fitting a carbon nanotube Raman spectrum" section), giving a decisive preference to the Murnaghan model, and the value of *b* it returns, 11.6 ± 0.3. Note the good agreement with the value from Perlin et al.^29^ If additionally we fix ${\mathrm{B}}^{\prime}$ at its literature value of 4.49^31^, lnBF is scarcely improved, because the Occam factor against this parameter is small, but the uncertainty on the pressure coefficient, Ξ/*B*_0_, is much improved.

When we fit the Perlin data, the Murnaghan fit returns ${\text{B}}^{\prime }$ = 6.6 ± 2.4. This is outside range, and indicates that this data cannot give a reliable value—attempting it is over-fitting. However, it is good to fit this data together with the Goñi data. The Perlin data, very precise but at low pressures only, complement the Goñi data with their lower precision but large pressure range. We notice also that the Perlin data has a proportion of outlier data points. Weighted or rescaled LS fitting can handle the different precisions, but it cannot handle the outliers satisfactorily. Maximum Likelihood fitting handles both issues. We construct ln*L* using different pdfs *P*(*r*) for the two datasets, and with a double-Gaussian pdf for the Perlin data (see equation (S6) in the SI §7). Fixing ${\text{B}}^{\prime }$ at 4.49, fitting with the same Ξ/*B*_0_ returns 11.42 ± 0.04 meV kbar^−1^. Separate Ξ/*B*_0_ parameters for the two datasets give an increase of ln*L* of 4.6, with values 11.28 ± 0.06 and 11.60 ± 0.04 meV kbar^−1^—a difference in *b* of 0.32 ± 0.07 meV kbar^−1^, which is significant at 4½σ. This difference could be due to systematic error, e.g. in pressure calibration. Or it could be real. Goñi et al.^28^ used absorption spectroscopy to measure the band-gap; Perlin et al.^29^ used photoluminescence. The increase of the electron effective mass with pressure might give rise to the difference. In any case, it is clear that high-pressure experimentation is much more accurate than previously thought, and that ML fitting exploits the information in the data much better than LS fitting.

### Background in fitting a carbon nanotube Raman spectrum

This example demonstrates how the Bayes Factor provides a quantitative answer to the problem, whether we should accept a lower quality of fit to the data if the parameter set is intuitively preferable. It also provides a simple example of a case where the MLI calculated by Eq. (1) is in error and can readily be corrected (see SI §8 Fig. S3).

**Figure 3:**
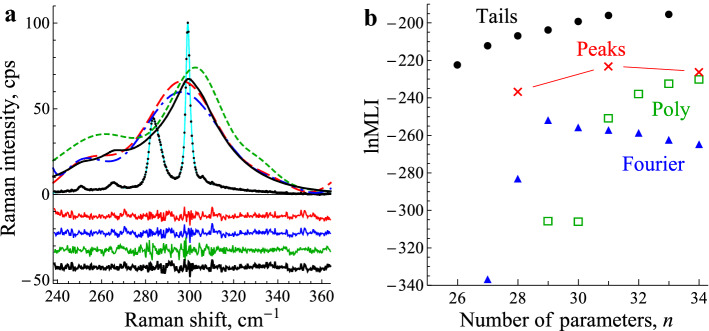
Carbon nanotube Raman spectrum. In (**a**), the carbon nanotube Raman spectrum is plotted (black datapoints) with a fit (cyan solid line) using the Fourier model. The residuals for four good fits are shown, × 10 and displaced successively downwards (Fourier, Polynomial, Peaks and Tails; all at ln*L* about − 60, see text). The backgrounds are shown, × 8 (long dashed, chain-dotted, short dashed and solid, respectively. In (**b**), the evolution of the MLIs is shown against the number of parameters for these four models. (Figure prepared using Mathematica 12.0, www.wolfram.com/mathematica/).

The dataset is a Raman spectrum of the radial breathing modes of a sample of carbon nanotubes under pressure^32^. The whole spectrum at several pressures is shown with fits in Fig. 1 of Ref.^32^. The traditional fitting procedure used there was to include Lorentzian peaks for the clear peaks in the spectra, and then to add broad peaks as required to get a good fit, but without quantitative figures of merit and without any attempt to explain the origin of the broad peaks, and therefore with no constraints on their position, widths or intensities. The key issue in the fitting was to get the intensities of the peaks as accurately as possible, to help understand their evolution with pressure. Here, we take a part of the spectrum recorded at 0.23 GPa (the data is given in the SI.) and we monitor the quality of fit and the Bayes factor while parameters are added in four models. This part of the spectrum has seven sharp pseudo-Voigt peaks (Fig. 3a; the two strong peaks are clearly doublets). With seven peak positions *P*_*i*_, peak widths *W*_*i*_ and peak intensities *A*_i_, and a factor describing the Gaussian content in the pseudo-Voigt peak shape, there are already 22 parameters (for details, see SI §8). This gives a visibly very poor fit, with ln*L* = − 440, SBIC = − 510 and lnMLI = − 546. The ranges chosen for these parameters for calculating the MLI (see SI §8) are not important because they are used in all the subsequent models, and so they cancel out in the Bayes factors between the models.

To improve the fit, in the Fourier model we add a Fourier background $y=\sum {c}_{i}\mathrm{cos}ix+{s}_{i}\mathrm{sin}ix$ (*i* = 0,..) and in the Polynomial model, we add $y=\sum {a}_{i}{x}^{i}$ (*i* = 0,..) for the background. In both, the variable *x* is centred (*x* = 0) at the centre of the fitted spectrum and scaled to be ± π or ± 1 at the ends. In the Peaks model we add extra broad peaks as background, invoking extra parameter triplets (*P*_*i*_, *W*_*i*_, *A*_*i*_). These three models all gave good fits; at the stage shown in Fig. 3a they gave ln*L* values of − 65, − 54 and − 51 and BIC values of − 156, − 153 and − 148 respectively. Thus there is not much to choose between the three models, but it is noteworthy that they give quite different values for the intensities of the weaker peaks, with the peak at 265 cm^−1^ at 20.5 ± 1.1, 25.5 ± 1.3 and 27 ± 1.7 respectively (this is related to the curvature of the background function under the peak). So it is important to choose wisely.

A fourth model was motivated by the observation that the three backgrounds look as if they are related to the sharp peaks, rather like heavily broadened replicas (see Fig. 3a). Accordingly, in the fourth model, we use no background apart from the zeroth term *c*_0_ or *a*_0_ to account for dark current). Instead, the peak shape is modified, giving it stronger, fatter tails than the pseudo-Voigt peaks (Tails model). This was done by adding to the Lorentzian peak function a smooth function approximating to exponential tails on both sides of the peak position (for details, see SI §8) with widths and amplitudes as fitting parameters. What is added may be considered as background and is shown in Fig. 3a. This model, at the stage of Fig. 3a, returned ln*L* = − 62, BIC = − 146, and yet another, much smaller value of 15.5 ± 1.0 for the intensity of the 265 cm^−1^ peak.

The Tails model is intuitively preferable to the other three because it does not span the data space—e.g. if there was really were broad peaks at the positions identified by the Peaks model, or elsewhere, the Tails model could not fit them well. That it does fit the data is intuitively strong evidence for its correctness. The Bayes factor confirms this intuition quantitatively. At the stage of Fig. 3a, the lnMLI values are − 251, − 237 and − 223 for the Fourier, Poly and Peaks models, and − 211 for the Tails model. This gives a lnBF value of 12 for the Tails model over the Peaks model—decisive—and still larger lnBF values for these models over the Fourier and Poly models.

All models can be taken further, with more fitting parameters. More Fourier or polynomial terms or more peaks can be added, and for the Tails model more parameters distinguishing the tails attached to each of the seven Lorentizian peaks. In this way, the three background models can improve to a ln*L* ~ − 20; the Tails model does not improve above ln*L* ~ − 50. However, as seen in Fig. 3b, the MLIs get worse with too many parameters, except when over-fitting occurs, as seen for the Poly model at 35 parameters. The Tails model retains its positive lnBF > 10 over the other models.

The other models can have an indefinite number of additional parameters—more coefficients or more peaks, to fit any data set. It is in this sense that they span the data space. The actual number used is therefore itself a fitting parameter, with an uncertainty perhaps of the order of ± 1, and a range from 0 to perhaps a quarter or a half of the number of data points *m*. We may therefore penalise their lnMLIs by ~ ln 4* m*^*−*1^ or about − 5 for a few hundred data points. This takes Tails to a lnBF > 15 over the other models—overwhelmingly decisive. This quantifies the intuition that a model that is not guaranteed to fit the data, but which does, is preferable to a model that certainly can fit the data because it spans the data space. It quantifies the question, how much worse a quality of fit should we accept for a model that is intuitively more satisfying. Here we accept a loss of − 30 on ln*L* for a greater gain of + 45 in the Occam factor. It quantifies the argument that the Tails model is the most worthy of further investigation because the fat tails probably have a physical interpretation worth seeking. In this context, it is interesting that in Fig. 3a fat tails have been added only to the 250, 265 and 299 cm^−1^ peaks; adding fat tails to the others did not improve the fit; however, a full analysis and interpretation is outside the scope of this paper. In the Peaks model it is not probable (though possible) that the extra peaks would have physical meaning. In the other two models it is certainly not the case that their Fourier or polynomial coefficients will have physical meaning.

## Discussion and conclusions

The most surprising outcome of "Examples of fitting data" section is the desirability of including in models some parameters that fail significance tests, and reporting the outcomes. This is relevant to the controversy about significance tests such as *p* values.

In the story of Mr A and Mr B, the two models are explicitly given equal a priori probabilities, *p*(**A**) = *p*(**B**) = ½ if there are no other models in contention, and before any data is considered the lnBF between them is zero. Suppose that the fit using model **A** has given a set of parameter values **V**_A_ = (*p*_*i*0_ ± δ*p*_*i*_), defining the posterior parameter volume. With model **B**, including the extra parameter, correlations between parameters result in giving $${\mathbf{V}}_{\mathrm{B}}=({p}_{i0}^{^{\prime}}\pm\updelta {p}_{i0}^{^{\prime}},{\uplambda }_{0}\pm \mathrm{\delta \lambda })$$, defining a different posterior parameter volume. The uncertainties $$\updelta {p}_{i}^{^{\prime}}$$ will generally be larger than δ*p*_*i*_, and the values $${p}_{i0}^{^{\prime}}$$ will generally be different from *p*_*i*0_. For illustration, suppose that λ_0_ is non-zero but fails significance tests, being perhaps just 1 or 2σ away from zero, and that the MLIs come out equal (i.e. the improvement in ln*L* in Model **B** is offset by the Occam factor, and lnBF remains at zero). Now to reject λ and to report only the fit to model **A** is to assert that the true values *p*_*i*_ have each a $${\raise0.5ex\hbox{$\scriptstyle 2$} \kern-0.1em/\kern-0.15em \lower0.25ex\hbox{$\scriptstyle 3$}}$$ chance of lying within **V**_A_, within the 1σ ranges δ*p*_*i*_. However, that assertion is conditional on λ actually having the value zero; that is, it is conditional on the truth of the null hypothesis **A**. And that is a condition that we do not know to be true. The failure of **B** to attain significance is often mistakenly described as evidence for the null hypothesis **A**. Amrhien et al. report that around half of a large number of articles surveyed in five major journals make this mistake^33^. It is not just a scientific mistake^10^. It can be a disastrous guide to action.

According to the Bayes factor, the models **A** and **B** have equal probabilities, $${\raise0.5ex\hbox{$\scriptstyle 1$} \kern-0.1em/\kern-0.15em \lower0.25ex\hbox{$\scriptstyle 2$}}$$, and so what we know is that the parameters of model **A** have each a $${\raise0.5ex\hbox{$\scriptstyle 1$} \kern-0.1em/\kern-0.15em \lower0.25ex\hbox{$\scriptstyle 3$}}$$ chance of lying within their 1σ ranges δ*p*_*i*_ around *p*_*i*_ and a $${\raise0.5ex\hbox{$\scriptstyle 1$} \kern-0.1em/\kern-0.15em \lower0.25ex\hbox{$\scriptstyle 3$}}$$ chance of lying within the 1σ ranges $$\updelta {{p}^{\mathrm{^{\prime}}}}_{i}$$ around $$p^{\prime}_{i}$$. In fact, in this situation (and especially if a significant non-zero λ_0_ would be an exciting result—see Ref.^34^ and discussion below for a current example) the usual reaction to finding that λ_0_ is 2σ away from zero is to repeat the experiment, to take more data. Of course, that has some chance of finding a λ_0_ closer to zero, but it also has a good chance of confirming a non-zero λ_0_. So the Bayes factor is a guide to action; the significance test is not.

Truth is not within the remit of probability theory. From its origins in Pascal’s and Fermat’s advice to the gambler the Chevalier de Méré (1654)^35^, probability is fundamentally about how to act when we do not know what will happen (or what is true), whether it be the turn of a card in poker, the weather forecast, or the administration of an untried medicament. We can write the value or profit of a potential success or win that has the probability *P*(win) as *V*(win) = *P*(win) × winnings, and similarly for a potential failure or a loss. In poker, the Expected Value of an action is defined as EV = *V*(win) − *V*(loss), and it is used to guide decisions how to act—whether to bet, or fold. The Bayes factor is the ratio of the probabilities of competing theories given the data. So it lends itself directly to multiplication by the financial or other quantifiable valuations of outcomes to guide actions.

Consider the current controversy about vitamin D and Covid-19. Model **A** (the null hypothesis) recommends inaction (action A), Model **B** recommends mass medication with vitamin D as a prophylactic (action B), and further research on the question (action C) may also be considered. The evidence for Model **B** is weak, but it is not insubstantial. A recent editorial in the BMJ concluded that it is strong enough to make the case for action C “compelling.”^36^ Martineau summarised the case for action B as “... it’s not the highest level of evidence. I guess there’s a philosophical question—if you have an intervention [action B] that has a good chance of working and is completely safe, why not implement it?”^37^.

Of course, there are answers to Martineau’s seemingly rhetorical question. There is the cost. Paying for action B means that something else won’t be paid for, and if that would have worked and action B does not then action B will—at least in hindsight—have been a poor decision. There is the question, which of perhaps an unlimited number of equivalent actions ${\text{B}}^{\prime }$ might be chosen—intravenous bleach, homeopathy or Vitamin D? If one, why not all the others? Martineau’s “if completely safe” is also important, since virtually nothing is completely safe. These points are important complexities, but citing them does not definitively answer the question.

Using the Bayes factor, Martineau’s question can be answered quantitatively. A “good chance” implies a lnBF in the range 1–2 for Model **B** against Model **A**. Crudely, the benefit of taking no action, A, is the saving on the cost of actions B and C. Maybe some £10^8^. The benefit of action B at once, if Model **B** is true, is, crudely, some £10^11^ in the avoidance of unnecessary deaths and lockdowns. The benefit of action C alone is much more complex, even negative, if it displaces research into other therapies, but, crudely, it delays action B so its best return is smaller. So the contributions of ln *V*(**B**)/*V*(**A**) to add to lnBF are about ln1000 =  + 7 for B and (less certainly) about + 5 for C alone. A full analysis should of course refine these costs and benefits by costing the complexities. And of course it could use other quantitative data than financial, such as numbers of deaths. But if it were to confirm these outcomes, both B and C should be undertaken urgently.

The issue of bleach and homeopathy is readily dealt with. With an unlimited number of putative actions ${\text{B}}^{\prime }$ based on models ${\mathbf{B}}^{\prime }$ to consider, their a priori probabilities should be rated as very small, except when there is evidence for them that is rated as not insubstantial. Then the factor *p*(${\mathbf{B}}^{\prime }$)/*p*(**A**) will outweigh—negatively—the factor *V*(${\mathbf{B}}^{\prime }$)/*V*(**A**).

For a simpler example, consider the example of Ref.^33^. They find evidence (from the LHCb experiment at CERN) for the violation of lepton universality (Model **B**), at the 3.1 sigma level (less than the 5-sigma level demanded in particles physics research), that is, a probability of 0.997, and a lnBF against the null hypothesis (Model **A**) of − ln 0.003 = 6. This is sufficient to encourage further work. It may be further increased by ln *V*(**B**)/*V*(**A**), if the value of physics beyond the Standard Model can be estimated, and the costs of the further work. The value is presumably of the order of the total cost of the Large Hadron Collider, as this is what is was built to find. The costs of some further work must be orders of magnitude less, so ln *V*(**B**)/*V*(**A**) may be about 5, increasing lnBF to 11, decisively in favour of further work.

In conclusion, calculation of Bayes factors should be a routine part of all data fitting. It gives advice that is the opposite of much standard practice, but which satisfies Occam’s Razor intuitions, and enables robust model selection and parameter estimation. Bayes factors, being the ratio of probabilities, are readily multiplied by financial or other quantitative data to quantify intuitive or philosophical arguments for actions.

## Supplementary Information

Supplementary Information 1.

Supplementary Information 2.
