# Supplementary material for: Easy computation of the Bayes factor to fully quantify Occam’s razor in least-squares fitting and to guide actions
Source: Sci Rep. 2022 Jan 19;12:993. doi: 10.1038/s41598-021-04694-7 (PMC8770492; doi:10.1038/s41598-021-04694-7)
Supplement: Supplementary file 1 — Supplementary Information 1. [file 41598_2021_4694_MOESM1_ESM.pdf]

Easy computation of the Bayes Factor to fully quantify Occam's razor

D.J. Dunstan,* J. Crowne, and A.J. Drew

School of Physics and Astronomy, Queen Mary University of London, London E1 4NS, UK.

Supplementary Information

§S1. Data: The data used in the three examples is given in the Supplementary Dataset spreadsheet Supplementary_Data.xlsx.

The muon data of Section 4.1 was provided as the data plotted in Fig.2a of Ref.27, with estimated uncertainties (mostly ± 0.15). Here we obtain best-fit residuals with a standard deviation of 0.008 at the p=10 fit and 0.004 at the p = 19 fit.

The GaAs data for $E_g(P)$ in Section 4.2 was digitised from journal Figures. For the Goñi *et al.*[28] data shown in their Fig.2, we re-digitised the data from Fig.1 of Ref.30. The data from Perlin *et al.*[29] was digitised from their Fig.9a (for 300K).

The carbon nanotube Raman spectrum of Section 4.3 came from the 0.23 GPa spectrum of the dataset presented in the PhD thesis of A.C. Torres-Dias (Université de Lyon, 2014). A.C. Torres-Dias *et al.*, Carbon **95**, 442 (2015) give details of the sample preparation and the identification of the peaks in the spectrum (their Fig.7a) and fits to the data as a function of pressure were reported in Ref.32, Fig.1, in which the y-ordinate was labelled as cps. The files provided to us were the files used for those fits. This file is clearly not raw, as there are some negative values of y, which cps (counts per second) cannot be. Perhaps a dark current has been subtracted.

§S2. Least-squares and Likelihood: Let the probability (pdf) that a residual is of magnitude r be Gaussian,

$$\begin{aligned} P(r) &= \frac{1}{\sqrt{2\pi\sigma^2}} e^{-\frac{r^2}{2\sigma^2}} \\ L &= \prod_{i=1}^{n} P(r_i) \\ \ln L &= -n\ln\frac{1}{\sqrt{2\pi\sigma^2}} - \sum_{i=1}^{n} \frac{{r_i}^2}{2\sigma^2} \end{aligned} \qquad \text{(S1)}$$

so that when $P(r)$ is Gaussian, as least-squares fitting assumes, minimising the sum of the squares of the residuals is the same thing as maximising L or ln$L$. Any other pdf $P(r)$ can be used, but then ML methods have to be used in place of LS.

§S3. Parameter Covariance Matrix: Some least-squares fitting routines return the parameter covariance matrix **Cov**. When that is not so, it can be readily calculated. The residuals are expressed as functions of the parameters p_i, and then the likelihood L can be calculated (§S2). Then the fitted values p_{i0} for all parameters except one, p_j, or two, p_j and p_k, can be substituted in to give the m functions $L(p_j)$ and the ½($m^2 - m$) functions $L(p_j, p_k)$. From these, the second derivatives can be calculated quite quickly. The Hessian matrix is

$$\mathbf{H} = \begin{pmatrix} \frac{\partial^2 L}{\partial p_1^2} & \cdots & \frac{\partial^2 L}{\partial \mathrm{p}_1 \partial p_m} \\ \cdots & \cdots & \cdots \\ \frac{\partial^2 L}{\partial p_1 \partial p_m} & \cdots & \frac{\partial^2 L}{\partial p_m^2} \end{pmatrix} \qquad \text{(S2)}$$

and then **Cov** = –$\mathbf{H}^{-1}$.

§4. Ranges of parameters: Ranges should be set *a priori*, using all knowledge that may be available, but before any inspection of the data. No great precision is needed, since it is the logarithms of the ranges that matter for lnBF. Choosing the ranges has been criticized as introducing subjectivity; however, the key point is that the ranges are given quantitatively and should be justified.[2,15,17] The values used can be defended or opposed on objective grounds. For physically-meaningless parameters such as polynomial coefficients, it is helpful to consider the data rescaled to the ranges 0 to 1 in x and y (that can be done automatically without inspecting the data). For example, if some data are to be fitted with $a + bx + cx^2$ and there is no prior information about the parameters, when the data are rescaled to the ranges 0 – 1 on both axes, all parabolae that could fit in this would be encompassed by the ranges $0 \leq a \leq 1$, $-4 \leq b \leq 4$ and $-4 \leq c \leq 4$. The rescaled data can be fitted and these ranges used, or these ranges can be rescaled for the original data. For the real examples of Section 4, see §§6-8 below.

§5. Theory of Eq.2: Figure S1 shows why the square-root of the determinant of the parameter covariance matrix in Eq.2 is used in place of the product of the parameter uncertainties. Data from Fig.2 is shown in Fig. S1a with the LS fit using $\Delta E_g = a_0 + bP + cP^2$ (a is fixed at its fitted value a_0 to make a two-parameter problem). The Eqn.S5 fit is also shown as the difference between the two fits is clearer here than in Fig.2. The MLI comes from the integral of L over the parameter space. From the LS fit, we have the fitted values b_0 and c_0 at which $L = L_{\max}$. We write L as a function of the residuals with b and c as variables, and it is easy to calculate a few values of L with either b or c varying and the other at its fitted value. These values are plotted in Fig.S1b, normalised to unity peak height, for parameter $p = b$ and for $p = c$, and with the x-axis normalised by the uncertainties σ_b and σ_c which are returned by the LS fit. These functions are accurate Gaussians, justifying the use of the Laplace approximation.

Notice that the widths of the two Gaussians of Fig.S1b, δb and δc, are much less than the uncertainties σ_b and σ_c by which the x-axis is normalised. Using the product $\sigma_b\sigma_c$ will give a gross overestimate of the two-dimensional integral of L. Equally, using the product $\delta b\delta c$ will give a gross underestimate. Plotting the full function $L(b, c)$ (Fig.S1c) shows why this happens. The function is Gaussian about its peak in all directions, but it is skew to the axes. The skewness expresses the parameter covariance. If the random errors in the data were to put, e.g., the initial gradient b higher, c will be fitted with a higher value as well. This corresponds to positive off-diagonal elements in the correlation and covariance matrices. To obtain the volume of $L(b, c)$, we need the area of the elliptical contour shown in Fig.S1c. The contour map of $L(b, c)$ (Fig.S1d) shows that $\sigma_b\sigma_c$ gives the area of the circle radius 1, and that using $\delta b\delta c$ instead gives the area of the small circle of radius 0.21.

The correct estimate can be found by taking linear combinations of the parameters p_i corresponding to a rotation in parameter space, such that the new parameters v_i will have zero correlation or off-diagonal covariance – that is, to change the parameter basis set. See Sec.2.3 and Eq. 2.56 in Ref.21. These new parameters are found by diagonalising the parameter covariance matrix. (If the fitting routine used does not return the parameter covariance matrix, it is not difficult to calculate; see SI §S3). The resulting eigenvectors specify the v_i.

Repeating the LS fit with these parameters, we get the identical fit and we can recover the original b_0 and c_0 by a back transform from the fitted values of v_i. Plotting $L(v_1, v_2)$ in Fig.S1e (contour plot in Fig.S1f) we see that now the product of the uncertainties $\sigma_{v_1}\sigma_{v_2}$ is the same as the product $\delta v_1 \delta v_2$ of the widths of the Gaussians $L(v_{1f}, v_2)$ and $L(v_1, v_{2f})$ and either gives us exactly the integral of L over the parameter space that we require.

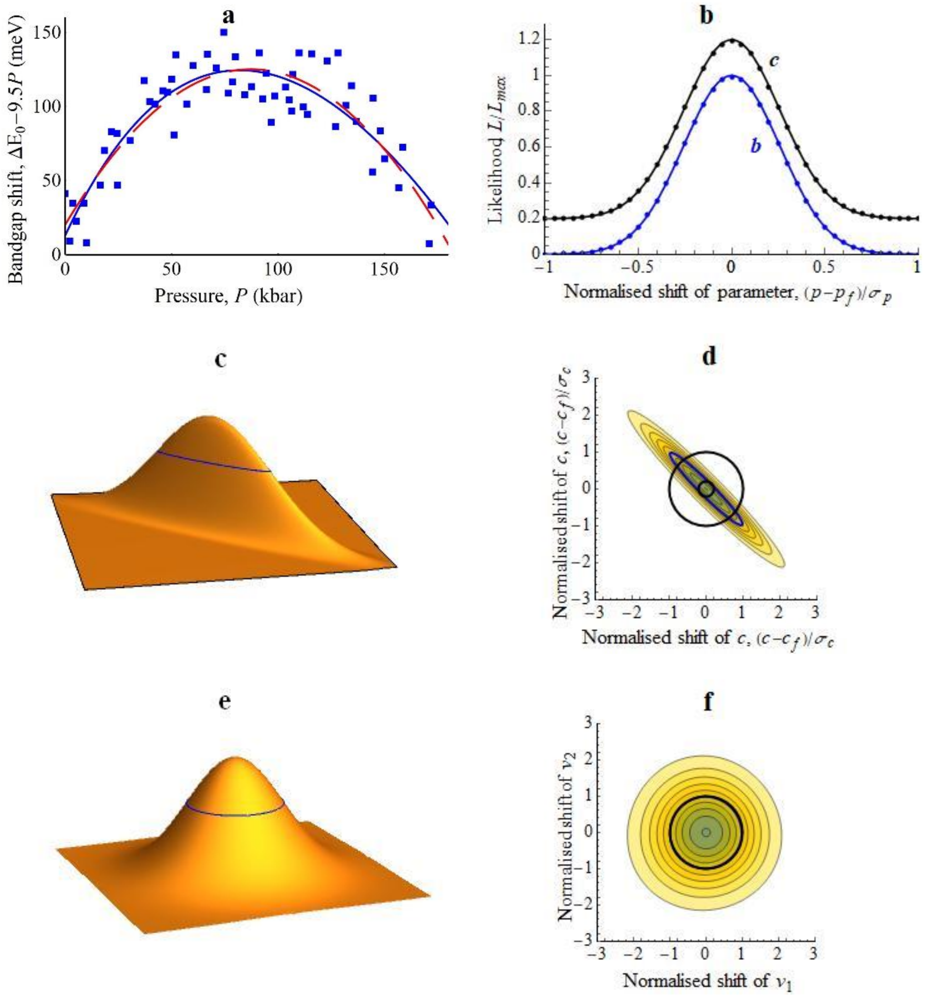

Fig.S1. In (a), data from Fig.2 ($E_g(P)$ of GaAs from Goñi *et al.*[16]) are shown after subtraction of the straight line $a_0 + 9.5P$ to make the curvature more visible. Two least-squares fits are shown, quadratic (dashed red line) and Eq.S5 (solid blue line). In (b), the likelihood of the quadratic fit is plotted as a function of the polynomial parameters p_i ($i = b, c$) scaled by their uncertainties σ_i, and fitted with Gaussians of width δb and δc. For clarity the data and fit for c are shifted up by 0.2. In (c) and (d), $L(b, c)$ is plotted with normalised axes, and the heavy blue ellipse is the contour at the height $e^{-½}$. On the contour plot in (d), the area of the large circle of radius 1 corresponds to the product of the uncertainties $\sigma_b\sigma_c$. Rotating the parameter basis set to v_1 and v_2 gives the corresponding plots of (e) and (f) from which the normalised products $\sigma_{v_1}\sigma_{v_2}$ and $\delta_{v_1}\delta_{v_2}$ are equal and both correctly give the area of the contour line. (Figure prepared using Mathematica 12.0, www.wolfram.com/mathematica/.)

To complete the calculation of the MLI with v_1 and v_2, we would need their parameter pdfs in parameter space. It is usual to estimate these as top-hat or rectangle functions, of width Δp_i and area unity. This is justified as a consequence of Laplace's Principle of Indifference.[15] Then the MLI is the integral of L that we have just found, divided by the product of the parameter ranges, $\Delta v_1 \Delta v_2$. However, we do not need to estimate the ranges Δv_i if we have already estimated the ranges Δb and Δc, because the product of the ranges is an area in either parameter basis, and areas are invariant under rotation. Better still, we do not need to make this transformation of the basis set nor to carry out the fit in this basis. The diagonal elements of the parameter covariance matrix in this basis, Cov_v, are the squares of the uncertainties on the parameters, so what we require for the calculation of the MLI in this basis is the product of these elements. Since the off-diagonal elements are zero, this product is also the determinant of Cov_v. But the determinant of a matrix is invariant to rotations, and so it is sufficient to take the determinant of the parameter covariance matrix in the p basis, Cov_p. That leads to Eq.2 for the MLI.

It is useful to compare what we do with Eq.2 with what is commonly done in the bulk of the Bayesian literature. There, L is expressed as an analytic function of the fitting parameters p_i, multiplied by the prior probability distribution over parameter space, and integrated. Because of the multi-dimensional nature of parameter space, this integration is done numerically using sampling methods. That gives the MLI directly. The position and the shape of L_{max} can be identified from the sampled values to give the parameter values and their uncertainties.

For the ML method, we also use $L(p_i)$. We could (but do not here) multiply it by a non-top-hat prior probability distribution. Instead of integrating it directly, we use the FindMaximum to obtain L_{max} and we construct the Hessian matrix at that point to give the shape of $L(p_i)$ around that point. We convert the Hessian to the covariance matrix, although that is not strictly necessary. There is sufficient information to evaluate the integral by the Laplace approximation, and, for a top-hat prior probability distribution, dividing by the prior parameter volume gives the MLI.

For the least-squares method, everything is strictly equivalent if the model, the hypothesis, has Gaussian-distributed residuals. The fitted parameter values and their uncertainties are returned at once and the covariance matrix is often returned too. So there is again sufficient information to evaluate the integral by the Laplace approximation, and, for a top-hat prior probability distribution, dividing by the prior parameter volume gives the MLI.

These three methods all follow the same logic. The only difference is how the arithmetic is done, what level of approximation is used in it, and what constraints may be placed on or implied for the models by the method (such as Gaussian residuals for least-squares).

Then we also use the parameter ranges to determine the top-hat prior probability density, in order to quantify the other Occam's razor intuitions that we discuss. This is not commonly done in the bulk of the Bayesian literature, nor is it done if the BIC is used.

§6. Example 1: Muon data fits.

Lorentzian peaks were used, with peak positions P_i, widths W_i and areas A_i. Light-induced effects were added by adding to each parameter p a term $z\delta_L p$, where $\delta_L p$ is the change in p under illumination, and writing the data in 3D as $y_i(x_i,z_i)$ with $z_i = 0$ for data in the dark and $z_i = 1$ for the photoexcited data. This enabled least-squares fitting to all data simultaneously, using the Mathematica function `NonlinearModelFit[data, func, {x,z}]`. Linear baselines were added, defined by the four corner points, y_{00} at $(x_{lo},0)$, y_{x0} at $(x_{hi},0)$, y_{0z} at $(x_{lo},1)$ and y_{xz} at $(x_{hi},1)$. The full model is thus,

$$y(x) = \sum_i \frac{A_i+z\delta_L A_i}{\pi} \frac{W_i+z\delta_L W_i}{(W_i+z\delta_L W_i)^2+(x-P_i-z\delta_L P_i)^2}$$

$$+(1-z)\big(y_{00}+y_{x0}(x-x_{lo})\big)+z\big(y_{0z}+y_{xz}(x-x_{lo})\big) \qquad \text{(S3)}$$

with the sum over i=1, i=1 to 2 and i = 1 to 3 for one, two and three peaks respectively. For i = 1, the parameters are not subscripted as here the data are all fitted by the single peak.

To calculate the MLI, ranges were set as follows. For the first peak, $\Delta P_1 = 400$ as it could be anywhere in the data. The second peak could not be far away as there was doubt whether one peak or two would be the best fit, so $\Delta P_2 = 100$. For widths and areas, $\Delta W = 50$ and $\Delta A = 40$ were chosen by considering the data range. Light-induced effects were expected to be small, so $\Delta\delta_L P = 100$, and for the widths and areas a 20% change was considered the most that could be expected, so $\Delta\delta_L W = 10$ and $\Delta\delta_L A = 8$. For the four background values, given that the positron background had already been subtracted, 20% of the data range was considered the largest plausible error in that subtraction, so $\Delta y_{ij} = 0.03$. When we add the third peak, its range $\Delta P_3 = 60$ is reduced because there isn't much room for it, $\Delta A_3 = 4$ because it has to be a weak peak, and accordingly $\Delta\delta_L A_3 = 1$. The other peak 3 parameter ranges were given the same values as for peaks 1 and 2.

§7. Example 2: Pressure dependence of the GaAs bandgap.

The Murnaghan equation of state derives from standard interatomic potentials and finite elasticity theory, using the approximation that the pressure derivative B' of the bulk modulus B_0 is constant. Then the equation for the volume as a function of pressure is

$$\frac{\Delta V}{V_0} = \left(1+\frac{B'P}{B_0}\right)^{-\frac{1}{B'}} - 1 \qquad \text{(S4)}$$

Assuming that the bandgap of GaAs, E_g, is linear with the density,[30]

$$\Delta E_g = \Xi\left[\left(1+\frac{B'P}{B_0}\right)^{\frac{1}{B'}} - 1\right] \qquad \text{(S5)}$$

where Ξ/B_0 is equivalent to b in the polynomial fit, $\Delta E_g = bP + cP^2$. For both fits we add a constant a to allow for uncertainty in the ambient-pressure value $E_g(P=0)$, so the quadratic fit has three terms and is denoted QP3.

The physical meanings of a and of $b \equiv \Xi/B_0$ are the same for all the fits. We choose $\Delta a = 100$ meV and $\Delta b = \Delta\Xi/B_0 = 5$ meV kbar^{-1} to be larger than the variations in the published fits. These values are not important as they cancel in comparisons of different models. $\Delta c = 100$ μeV kbar^{-2} and $\Delta B' = 1$ are chosen as described in Section 4.2.

ML fitting is used because the Perlin data shows outliers among the residuals after LS fitting (Fig.S2). For this, the pdf of the residuals for these data is written as the sum of two Gaussian distributions, and the likelihood is written as

$$L = \prod_{data_1} r_i \times \mathrm{pdf}_1 \times \prod_{data_2} r_j \times \mathrm{pdf}_2$$

$$= \prod_{i=1}^{88} \left(\frac{1-f}{\sqrt{2\pi\sigma_\mathrm{P}^2}} \exp\left(-\frac{r_i^2}{2\sigma_\mathrm{P}^2}\right) + \frac{f}{\sqrt{2\pi\sigma_\mathrm{O}^2}} \exp\left(-\frac{r_i^2}{2\sigma_\mathrm{O}^2}\right) \right)$$

$$\times \prod_{j=89}^{146} \frac{1}{\sqrt{2\pi\sigma_\mathrm{G}^2}} \exp\left(-\frac{r_j^2}{2\sigma_\mathrm{G}^2}\right) \qquad \text{(S6)}$$

where $data_1$ is the Perlin[29] data (datapoints i = 1 to 88) and $data_2$ is the Goñi[28] data (datapoints j = 89 to 146). The three Gaussians have σ_G for the Goñi data, σ_P for the good Perlin datapoints, and σ_O for the outlier Perlin datapoints. The fraction f may be interpreted as the fraction of the Perlin data that are outliers.

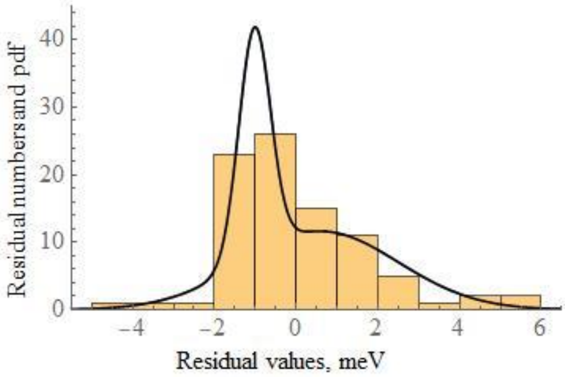

Fig.S2. A histogram of the residuals returned by the least-square fit to the Perlin *et al.*[28] data. The double Gaussian pdf used in the maximum-likelihood fitting of these data is plotted, multiplied by the number of data points (88). (Figure prepared using Mathematica 12.0, www.wolfram.com/mathematica/.)

Table S1. Fits as described in the text to the data of Goñi *et al.*[28] (Go) and Perlin *et al.*[29] (Pe). The methods are least-squares (LS) and maximum likelihood (ML). In the 4th column, the values are b for the QP3 fit and Ξ/B_0 for Eq.S5, in units of meV kbar^{-1}; in the 5th column c for the QP3 fit has units μeV^2 kbar^{-2}. B' is dimensionless; for it, 4.49* was fixed not fitted.

Model	Method	Data	b, Ξ/B_0	c, B'	ln$L$	SBIC	lnMLI	lnBF	Evidence
QP3	LS	Go	10.9 ± 0.2	–14 ± 1	–247.18	–255.34	–258.34	0	
Eq.S5	LS	Go	11.6 ± 0.3	4.5 ± 0.3	–247.17	–255.33	–254.55	3.8	Strong
Eq.S5	LS	Go	11.6 ± 0.1	4.49*	–247.17	–253.28	–254.40	3.9	Strong
Eq.S5	ML	Pe + Go	11.42 ± 0.04	4.49*	–417.6	–428.1	–429.9	0	
Eq.S5	ML	Pe	11.28 ± 0.06						
		& Go	11.60± 0.04	4.49*	–408.8	–421.6	–425.3	4.6	Strong

§8. Example 3. Nanotube Raman fit.

For the Raman data, Fig3, the ranges for the 23 parameters common to the three models were ΔP_i = 120 cm^{-1} for the Raman peak positions as they must fall in the range of the data, 238 to 364 cm^{-1}. For their widths, ΔW_i = 10 cm^{-1}, on physical grounds (we know they are nanotube RBM modes). For the areas, ΔA_i = 500 cm^{-1}, about twice the largest peaks present. The zero offset (dark count rate) is expected to be of the order of 1. The fraction of Gaussian in the Voigt lineshape, G, is necessarily between 0 and 1, so $\Delta G = 1$. In the Fourier and Poly models, the extra parameters were given ranges $\Delta c_i = \Delta s_i = \Delta a_i = 200$, except for c_0 and a_0 which represent the zero offset (dark count rate), expected to be of the order of 1, and so $\Delta c_0 = \Delta a_0 = 1$. For the extra peaks in the Peaks model, ΔP_i = 120 cm^{-1} again, and here and in the Tails model, ΔW_i = 100 cm^{-1} as these can be much broader than the sharp peaks. The intensities can be correspondingly large, for Peaks we used ΔA_i = 1000 cm^{-1}. For the Tails model, we gave the tail intensity for peak i as $f \times A_i$ and the fitting parameter f was given the range $\Delta f = 5$.

While we tried adding broad Lorentzian tails to each peak, obtaining at best ln$L$ = –80, the function

$$y = \frac{1}{2\gamma} e^{-\gamma^{-1}\sqrt{x^2+5}} \qquad \text{(S7)}$$

gave much better fits with the best ln$L$ = –50. This function has symmetrical exponential tails with a little rounding at $x = 0$ to make it continuously differentiable.

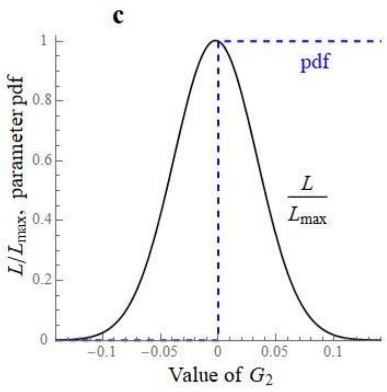

Fig.S3. The normalised likelihood function $L(G_2)$ (solid line) and the pdf of this parameter (dashed line) are plotted. (Figure prepared using Mathematica 12.0, www.wolfram.com/mathematica/.)

Finally, Fig.S3 shows a case earlier in the fitting, where the calculated Bayes Factor is in error but the error is readily detected and corrected. We gave each of the seven peaks its own fraction G_i of Gaussian content in the pseudo-Voigt peak shape. Fitted values were sometimes outside the meaningful range of $0 \leq G_i \leq 1$, so this condition was added as a constraint. Fig.3c shows that in this case, with a fitted value of $G_2 = 0$, half of the Gaussian function $L(G_2)$ is outside the range of non-zero probability for this parameter. Its contribution to the MLI integral is thus twice what it should be. This can be corrected by dividing the MLI by 2, or subtracting ln2 from the lnMLI and the lnBF.
